# Microsurgical reconstruction of the enormous traumatic oromandibular defect by osteocutaneous fibula-free flap in a 9-year-old child: a case report

**DOI:** 10.11604/pamj.2022.43.13.36738

**Published:** 2022-09-07

**Authors:** Abdulfattah Altam, Saleh Alredae, Ahmed Alsaaidi, Faisal Ahmed, Waleed Aljbri, Burkan Nasr, Qasem Alyhari, Abdullah Al-Nagga

**Affiliations:** 1 Department of General Surgery, School of Medicine, Sana´a University, Sana´a, Yemen; 2 Department of Maxillofacial and Dental Surgery, School of Medicine, Sana´a University, Sana´a, Yemen,; 3 Department of Urology, School of Medicine, Ibb University of Medical Sciences, Ibb, Yemen,; 4 Department of Urology, School of Medicine, Sana´a University, Sana´a, Yemen,; 5 Department of General Surgery, School of Medicine, Ibb University of Medical Sciences, Ibb, Yemen,; 6 Department of anesthesiology, Al-Thora Modern Hospital, Faculty of Medicine, Sana´a University of Medical Sciences, Sana´a, Yemen

**Keywords:** Oromandibular defect, children, microsurgery, osteocutaneous fibula-free flap, case report

## Abstract

Enormous oromandibular defects in children remain a reconstructive challenge due to the region's unique features and the scarcity of a perfectly matched recipient site. The osteocutaneous fibula-free flap (OCFFF) is an excellent option for these defects. Most reports on oromandibular reconstruction in children are limited to surgical techniques instead of long-term follow-up, especially in resource-limited settings. We reported a 9-year-old child who presented with a massive oromandibular defect caused by a high-energy gunshot. Firstly, the patient was treated with debridement, lower defect edges approximation, and tracheostomy. After one week, the procedure of OCFFF was performed, and two months later, the lower lip was reconstructed using a tongue flap. The aesthetic outcome was excellent at two years, and the patient could speak and eat without impaired oral function. In conclusion, microsurgical reconstruction using OCFFF for massive oromandibular defects in our child patient was safe with satisfactory facial aesthetics and oral function.

## Introduction

Enormous oromandibular defects can result from gunshot wounds, burns, or oncological resection that frequently result in severe facial deformities and disfigurement, making reconstruction difficult [1]. Augmented local flaps can provide a long-term solution for moderately facial defects, but in the case of damaged local tissues or extensive facial damage, distant tissue-free flap transfer is the best option [2]. The osteocutaneous fibula-free flap (OCFFF) has recently emerged as one of the more commonly used approaches due to its consistent, credible anatomy and diverse application areas, particularly in complex oromandibular defects where the lower lip, chin, and entire mandible are totally destructed [3]. There are few reports on oromandibular reconstruction in children. Most of these reports are limited to surgical techniques instead of long-term follow-up, especially in developing countries with resource-limited settings [4,5]. Hence, we report a successful microsurgical reconstruction of an enormous oromandibular defect in a 9-year-old pediatric patient using OCFFF.

## Patient and observation

**Patient information:** a 9-year-old child presented with severe lower face injury with subtotal mandibular destruction and a significant lower face soft tissue defect caused by a high-energy gunshot injury in January 2020.

**Figure 1:**
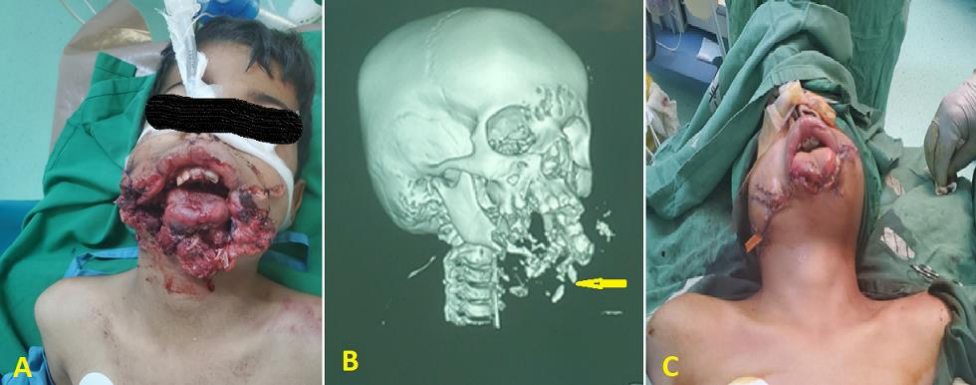
A) facial view at the time of initial evaluation; B) 3D reconstruction computed tomography scan showing subtotal destruction of mandibular bone (arrow); C) postoperative photo after approximation of the lower face defect edges

**Clinical findings:** in the initial evaluation, the patient was in shock status with a blood pressure of 70/50 mmHg, respiratory rate of 25 respirations per minute, and pales rate of 96 beats per minute. Physical examination revealed a loss of skeletal and soft tissue in the mid and lower face. There were massive lower face defects due to the loss of 21 cm of mandibular bone, its overlying soft tissue, the entire lower lip, the oral floor, and the lower surface of the anterior third of the tongue (Figure 1A).

**Diagnostic assessment:** the white blood cells: 11x103/ml, hemoglobin: 7g/L, and platelets count: 200x103/L. The other blood investigation tests were within normal limits, including liver function tests, coagulation tests, and renal function tests. The head and neck computed tomography (CT) scan relieved subtotal mandibular destruction (Figure 1B).

**Figure 2:**
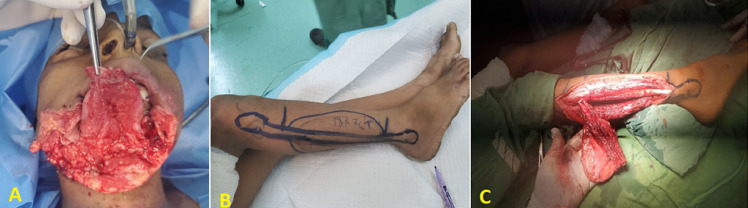
A) preoperative photo showing bone, soft tissue, and skin defects; B) intraoperative photo showing the location and preparation of osteocutaneous fibula-free flap; C) the osteocutaneous fibula-free flap after dissection and harvesting

**Figure 3:**
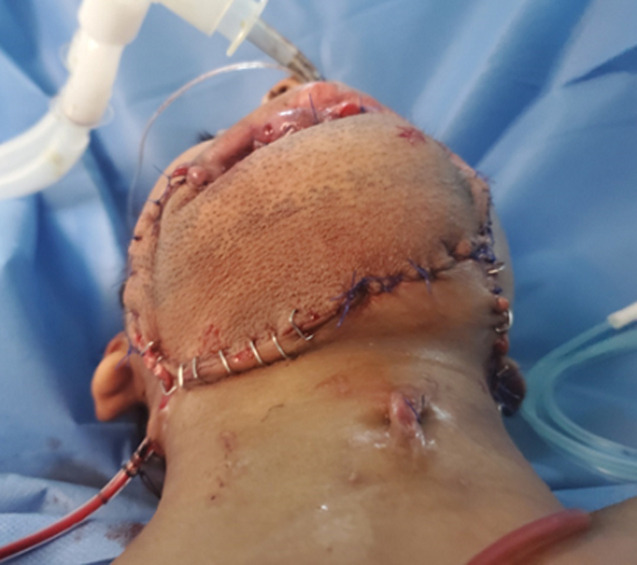
the results of flap coverage immediately after operation

**Therapeutic interventions:** the patient was urgently transferred to the operating room. After general anesthesia, resuscitation with one litter of normal saline, antibiotic therapy, and blood products was initiated, then initial debridement and approximation of the lower face defect edges and tracheostomy were performed as initial emergency procedures (Figure 1C). One week later, after stabilizing the patient´s condition, controlling the infection with appropriate antibiotics, and good enteral feeding via nasogastric tube, the reconstruction of the lower face using OCFFF was performed. Firstly, facial defects were cleaned and debrided. Then, the bone and soft tissue and skin defects were estimated (21 cm for bone and 15x6 cm for skin and soft tissue) (Figure 2A). The recipient´s vessels were prepared under loupe magnification, and the left external carotid artery, left internal jugular vein, and left external jugular vein was selected as the recipient´s vessels. Design and mark OCFFF at the patient´s right leg with bone length 21 cm, skin paddle 18x7 cm with aids of hand Doppler ultrasonography which was used to evaluate the site of skin perforators (Figure 2B). The OCFFF was harvested in the standard procedure described by Hidalgo (Figure 2C) [6]. The harvested OCFFF was transverse and fixed to mandibular stumps with multiple bicortical screws. Micro-anastomoses were made with one arterial and two veins anastomosis via interrupted 0.9 nylon sutures (Figure 3). The operative time lasted 10 hours, and no blood transfusion was needed. At the end of the operation, the flap was warm, with good capillary refilling with cherry red bleeding from the pen break point.

**Figure 4:**
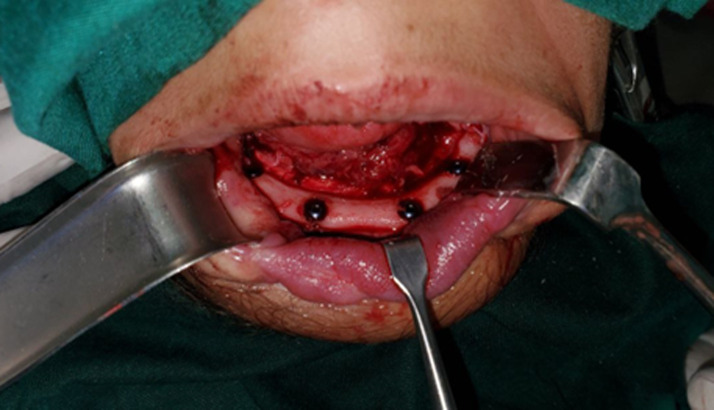
intraoperative photo showing the dental implantation

**Figure 5:**
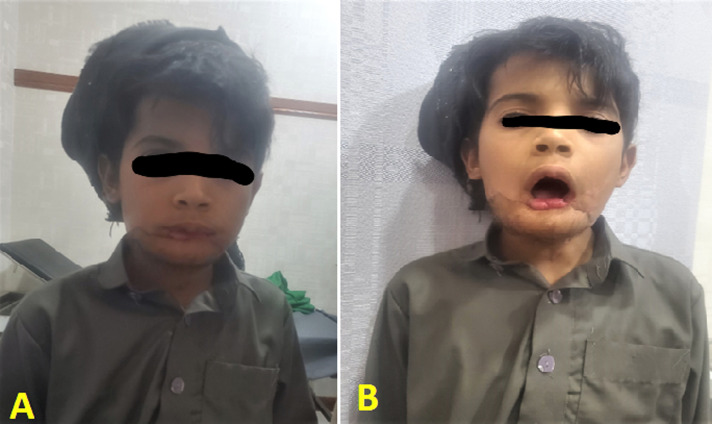
photos at two years of postoperative follow-up; A) with a closed mouth; B) with an open mouth

**Follow-up and outcome:** the patient was shifted to the intensive care unit (ICU) for continued flap monitoring by assessing flap skin color, surface temperature, rate of bleeding with pinprick or scratch, and capillary refill time. The patient received proper antibiotics (Clindamycin 30 mg/kg/day intravenously divided q8h and Gentamicin 2 mg/kg/dose intravenously every 8 hours for seven days), anticoagulant therapy was subcutaneously (enoxaparin 2000IU two times per day for seven days), and pain analgesic therapy. The oral liquid diet was started on the third postoperative day. The patient was transferred to the surgical ward on the 10^th^ postoperative day and discharged home on the 14^th^ postoperative day. Two months later, the patient was admitted for lower lip reconstruction with a tongue flap that divided three weeks ago. Five months later, the dental implantation was done for the lower jaw over the OCFFF (Figure 4). Follow-up at two years postoperatively, the mouth opening was more than 6 cm. The patient was able to eat a soft diet normally without impaired chewing. The patient´s speech was normal, and the aesthetic outcome was very good (Figure 5).

**Patient perspective:** the patient and his family were pleased with the care she received throughout therapy.

**Informed consent:** written informed consent was obtained from the patient family for participation in our study.

## Discussion

This case describes the microvascular free-flap procedure for the reconstruction of highly complex oromandibular defects in a 9-year-old child patient. With this concept, we were able to create a prefabricated reconstruction with proper oral function and good facial aesthetic outcomes using an OCFFF. Facial fractures in children are rare, with an approximate prevalence rate of 10%, more in boys, and the majority of these fractures occur at school age and in adolescence [7]. There are few reports on oromandibular reconstruction in children. Most of these reports are limited to surgical techniques instead of long-term follow-up, especially in developing countries with resource-limited settings [4,5,8]. According to facial gunshot injuries calcifications, our patient had a substantial facial defect representing a mixture of several forms [9]. Our patient was firstly treated with debridement, lower defect edges approximation, and tracheostomy. The initial procedure aims to debride necrotic tissues, perform a tracheostomy for injured airways, and treat intracranial injuries [7]. If tissue viability is in doubt during wound debridement, it is retained to declare itself within 24-72 hours. This is especially true of skin margins and tissues of the nose, palate, lips, and medial canthal areas, where tissue survival can significantly influence flap reconstruction choice or how a specific flap is used for definitive reconstruction [4]. The optimal time for the reconstruction of these injuries is still debatable. However, reconstruction during the subacute stages (2 to 3 days after the insult) was associated with a lower infection rate and other flap-related adverse events, allowing frequent debridement [10]. However, the clinical approach to severe oromandibular defects is to perform the ultimate treatment option in one stage to prevent the remaining soft tissue contraction. Such an effort can provide better aesthetic and functional outcomes, as performed by Nişanci *et al*. and in our patient [7].

The computed tomography (CT) scan is a good radiologic option to evaluate the pattern of oromandibular defects and displays the exact fracture pattern along with communication. Additionally, the digital configuration of the defect and fabrication of an individualized stereolithic mandibular model has been made possible by advances in computerized tomography scans [6]. Osteocutaneous fibula-free flap is the gold standard option for complex oromandibular defect reconstruction [7]. Osteocutaneous fibula-free flap is easily harvested, has good sculptability, and provides acceptable functional outcomes, as performed in our patient [11]. The anterolateral thigh flaps, iliac crest free flaps, and radial forearm free flaps are alternative options for soft tissue reconstruction [11,12]. The principle's surgical approach includes intraoperative mandibular segment debridement, defects' size evaluation, and surgical manipulations of the flap on the recipient site [7]. We used a fibula for bony reconstruction and the skin paddle for the mucosal defect. Similar to our technique, Krane *et al*. used a single OCFFF with a single pedicle to reconstruct complex facial defects [13]. In contrast, Nişanci *et al*. used a simultaneous transfer of three free flaps to reconstruct a complex facial skeletal and soft tissue defect resulting from a gunshot injury. The authors used a radial forearm flap to replace the internal lining and external cover of the nose, a large OCFFF restored the lower face, and a second OCFFF harvested from the other leg restored the midface [7]. Tissue quality is good in children, and complications such as nonunion, malunion, and infection are less common in these populations. However, unlike skeletally mature adults, pediatric patients will continue to develop local dynamic changes following flap transfer as they grow, raising concerns about free-bone flaps in this population [4,10]. Following OCFFF transfers, ankle instability and other donor-site mobilities are common complications. Because of the complexity of these deficits and the differential growth of the reconstructed and uninvolved sides, symmetry is an elusive goal in pediatric facial reconstruction [4,10]. The mandibular width grew significantly between one and five years and continued to grow between eight and twelve years. The mandibular height stops increasing at the age of five years. According to Zhang *et al*.´s review, more than 50% of the children aged less than eight showed impaired growth after reconstruction, whereas most children aged 8-12 years, during the rapid growth period, showed strong growth potential after reconstruction [8,14]. Our patient was nine years old and showed no impairment growth within two years of follow-up. Additionally, patients should be informed that future interventions to improve facial symmetry may be suggested [4]. Our patient´s aesthetic outcome was excellent at two years, and the patient could speak and eat without impaired oral function, without complication.

## Conclusion

The microsurgical reconstruction using OCFFF for massive oromandibular defects in our child patient was safe with satisfactory facial aesthetics and oral function. This method could be the foundation to apply to children´s patients with oromandibular defects in the future.
